# Prophylactic Fetal Creatine Supplementation Improves Post‐Asphyxial EEG Recovery and Reduces Seizures in Fetal Sheep: Implications for Hypoxic–Ischemic Encephalopathy

**DOI:** 10.1002/ana.27150

**Published:** 2024-12-07

**Authors:** Nhi T. Tran, Stacey J. Ellery, Sharmony B. Kelly, Juliane Sévigny, Madeleine Chatton, Hui Lu, Graeme R. Polglase, Rod J. Snow, David W. Walker, Robert Galinsky

**Affiliations:** ^1^ The Ritchie Centre, Hudson Institute of Medical Research Melbourne Victoria Australia; ^2^ Department of Obstetrics and Gynecology Monash University Melbourne Victoria Australia; ^3^ University of Sherbrooke Quebec Canada; ^4^ Department of Pediatrics Monash University Melbourne Victoria Australia; ^5^ Institute for Physical Activity and Nutrition Deakin University Melbourne Victoria Australia

## Abstract

**Objective:**

Hypoxic–ischemic encephalopathy (HIE) is a major cause of perinatal brain injury. Creatine is a dietary supplement that can increase intracellular phosphocreatine to improve the provision of intracellular adenosine triphosphate (ATP) to meet the increase in metabolic demand of oxygen deprivation. Here, we assessed prophylactic fetal creatine supplementation in reducing acute asphyxia‐induced seizures, disordered electroencephalography (EEG) activity and cerebral inflammation and cell death histopathology.

**Methods:**

Fetal sheep (118 ± 1 days’ gestational age [dGA]; 0.8 gestation) were implanted with electrodes to continuously record EEG and nuchal electromyogram activity. At 121 dGA, fetuses were randomly assigned to sham control (i.v. saline infusion without umbilical cord occlusion [UCO]; SalCon), continuous i.v. creatine infusion (6 mg/kg/h; CrUCO) or isovolumetric saline (SalUCO) followed by UCO at 128 ± 2 dGA that lasted until the mean arterial blood pressure reached 19 mmHg. Brain tissue was collected for histopathology after 72 hours of recovery.

**Results:**

Creatine supplementation had no effects on basal systemic or neurological physiology. UCO duration did not differ between CrUCO and SalUCO. After reperfusion, CrUCO fetuses had improved EEG power and frequency recovery and reduced electrographic seizure incidence (SalUCO, 86% vs CrUCO, 29%) and burden. At 72 hours after UCO, cell death in the cerebral cortex and astrogliosis in the periventricular white matter were reduced in CrUCO fetuses compared with SalUCO.

**Interpretation:**

Creatine supplementation reduced post‐asphyxial seizures and improved EEG recovery. Improvements in functional recovery with creatine were associated with regional reductions in cell death and astrogliosis. Prophylactic creatine treatment has the potential to mitigate functional indices of HIE in the late gestation fetal brain. ANN NEUROL 2025;97:673–687

Hypoxic–ischemic encephalopathy (HIE) is a major cause of neonatal mortality and life‐long neurological disability, affecting ~ 2 to 15 of 1,000 livebirths every year.^2^ In developed countries, mild therapeutic hypothermia (cooling) for HIE in near‐term and term infants improves survival without disability, however, current protocols are only partially protective such that ~ 30% to 50% of infants still die or survive with disability. Moreover, in low‐ and middle‐income countries (LMICs), the feasibility, safety, and efficacy of cooling remains uncertain.^3^ Thus, there is an important unmet need to identify other easily deployable and safe treatments that can be used to further reduce the incidence and severity of HIE globally.

Creatine has emerged as a promising neuroprotective agent for hypoxic–ischemic (HI) brain injury in the adult and developing brain.^4^ Creatine, and its phosphorylated form phosphocreatine, are nitrogenous guanidine compounds involved in spatial and temporal buffering of cellular adenosine triphosphate (ATP) during periods of high metabolic demand, and with the stress that occurs in the brain during oxygen deprivation.^5^ Therefore, creatine supplementation during pregnancy could offer prophylactic cerebral metabolic support to mitigate the pathophysiology of HIE, and more generally for the improvement of reproductive and maternal health.^6^ For example, in near‐term fetal sheep, intravenous (i.v.) fetal creatine infusion reduced the cerebral metabolic and oxidative stress responses to acute asphyxia.^7^, ^8^, ^9^, ^10^ Other potential therapeutic mechanisms linked to creatine include anti‐excitotoxicity, neuromodulation, and reduced cell death and gliosis in rodents, and also in *in vitro* models of hypoxic injury.^4^, ^11^, ^12^, ^13^

Electroencephalography (EEG) to assess brain function provides an ideal investigative tool to further our understanding of creatine for the treatment of HIE. Indeed, EEG features, such as frequency, amplitude (or voltage) of activity, organization of fetal behavioral states, background activity, and sleep‐state cycling (SSC), provide important functional insights relating to HIE prognosis.^1^ The highly integrated patterns of EEG activity present in late gestation in sheep, and in the third trimester of human pregnancies, that resemble “quiet” and “active” sleep arise from neural networks comprising the reticular activating system in the pons and diencephalon.^14^ Moreover, brief severe hypoxia impairs the coordination of fetal behavioral states,^15^ and increases seizure‐like activity that is associated with adverse neurodevelopmental outcomes.^16^, ^17^ Indeed, anticonvulsant effects of prophylactic creatine supplementation have been reported in multiple preclinical animal models of pentylenetetrazol‐induced seizures,^18^ and in humans with cerebral creatine deficiencies who are at increased risk of seizures.^19^

Despite perinatal studies showing biomolecular and biochemical benefits, and adult studies showing neuromodulatory and anti‐excitotoxic effects of creatine for hypoxic brain injury, to the best of our knowledge, the effects of creatine on the pathophysiology of perinatal HIE are yet to be rigorously tested using a clinically translatable experimental paradigm. In this novel study, we assessed the neuroprotective capacity of pretreatment with creatine on electroencephalographic markers of cerebral function and seizure activity in a preclinical model of mild to moderate HIE. Specifically, in late gestation fetal sheep, we investigated whether pretreating the fetus with creatine improved fetal physiological responses to acute asphyxia, with a focus on assessing seizures, EEG recovery, and fetal behavior.

## Methods

Animal experiments were approved by the Monash Medical Centre Animal Ethics Committee (MMCA/2019/23 and MMCA/2017/13). Animal handling, care, and use were conducted in accordance with the National Health and Medical Research Council (NHMRC) Australian Code of Practice for the Care and Use of Animals for Scientific Purposes (8th edition). The experiments are reported in accordance with the ARRIVE guidelines for reporting animal research.

### Fetal Surgery

**FIGURE 1:**
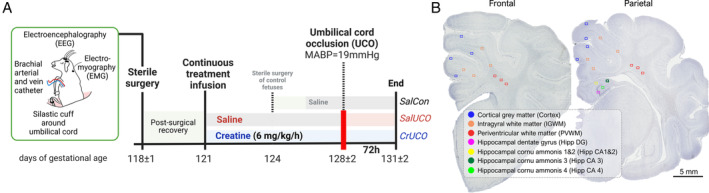
Experimental timeline and schematic diagram indicating fields sampled for histological assessment. (A) Experimental timeline and (B) fields of view of regions of interest assessed for histology. Immunohistochemistry was conducted on a rostral section of the frontal lobe and caudal section of the parietal lobe of the right cerebral hemisphere of the fetal sheep brain.

Pregnant Border‐Leicester ewes underwent sterile surgery between 118 and 119 days of gestation (dGA; see Supplementary Materials and Methods, and Fig 1A). Food but not water was withdrawn approximately 18 hours before surgery. Anesthesia was induced by i.v. injection of sodium thiopentone (20 mg/ml; 20 ml) and maintained using 2% to 3% isoflurane in oxygen (Bomac Animal Health, New South Wales, Australia). Ewes received prophylactic antibiotics (500 mg ampicillin, Austrapen; Alphapharm Pty, Australia; and 500 mg oxytetracycline hydrochloride, Engemycin‐100; Coopers Animal Health, Australia) immediately before surgery. Isoflurane levels, heart rate, oxygen saturation, and respiratory rate were continuously monitored throughout surgery by trained anesthetic staff. A midline laparotomy was performed, the fetus was exposed, and partially removed from the uterus. An inflatable silastic vascular occluder (ID 16 mm; In Vivo Metric, Healdsburg, CA, USA) was placed around the umbilical cord close to the fetal abdomen. The right brachial artery and vein were catheterized for serial arterial blood sampling and intravenous creatine/saline infusion, respectively. A catheter was secured in the amniotic sac to measure intra‐uterine amniotic pressure and maternal movement for arithmetic correction of fetal arterial blood pressure. Two pairs of EEG electrodes (AS633‐7SSF; Cooner Wire, Chatsworth, CA, USA) were placed onto the dura through 2 mm diameter cranial burr holes over the left and right parasagittal parietal cortex (10 and 20 mm anterior to bregma and 10 mm lateral) and secured to the skull with surgical bone wax and cyanoacrylate glue. EEG instrumentation allowed for measurements of brain activity (power and frequency), electrographic seizure detection, and analysis of fetal behavioral states. A pair of electrodes was sewn into the nuchal muscle to record electromyographic (EMG) activity as a measure of fetal movement and a reference electrode was sewn over the occiput. At the end of surgical instrumentation, the fetus was returned to the uterus, fetal leads were exteriorized through the maternal flank, surgical incisions were sutured closed, and the ewe and fetus were allowed to recover. All sheep were housed together in separate pens with access to food and water ad libitum in a temperature‐controlled room (18 ± 1°C, humidity 50 ± 10%) with a 12/12 hours light dark cycle.

### Experimental Protocol

#### Experimental Recordings

Continuous recordings of fetal mean arterial blood pressure (MABP), amniotic pressure, fetal heart rate (FHR; derived from the beat‐to‐beat interval of the brachial artery pulse), EEG, and nuchal EMG started at 120 dGA (24 hours before starting creatine/saline infusion) and continued until the end of the experiment (131 ± 2 dGA). All fetal physiological parameters were filtered and digitized using commercial hardware (Powerlab, ADInstruments, Australia) and recorded continuously using LabChart Pro software (version 8.1.16; ADInstruments, Australia; Supplementary Fig S1). EEG power was derived from the intensity spectrum signal using a bandpass filter with a cut off frequency set at 1 and 22 Hz and digitized at a sampling frequency of 400 Hz. Spectral edge frequency (SEF) was calculated as the frequency below which 90% of the intensity was present. Relative spectral power, expressed as the percentage of total frequency, in the delta (0–3.9 Hz), theta (4–7.9 Hz), alpha (8–12.9 Hz), and beta (13–22 Hz) frequency bands, was quantified by calculating the power spectra, by fast Fourier transform (FFT), of the EEG on sequential epochs using an FFT size of 32 K and a Blackman data window. For data presentation, total EEG power was converted into decibels (dB) using log‐transformation (10 × log_10_ intensity). The fetal nuchal EMG signal was band pass filtered between 100 Hz and 1 kHz, and the signal integrated and digitized at a sampling frequency of 2 Hz. A fetal arterial blood sample (1 ml) was taken each day between 09:00 and 10:00 hours to measure blood pH, PaCO_2_, PaO_2_, oxygen saturation (SaO_2_), glucose, lactate, base excess, and HCO_3_^−^ using an ABL90 Flex Plus analyzer (Radiometer, Denmark).

#### Intervention Groups

At 121 dGA, fetuses were randomly assigned, using an online number generator, to the following groups:

1. Fetal i.v. saline (0.9% NaCl, pH 7.4) + sham occlusion (SalCon, n = 7)
2. Fetal i.v. creatine monohydrate (dose: 6 mg/kg/h daily) + umbilical cord occlusion (CrUCO, n = 7)
3. Fetal i.v. saline + umbilical cord occlusion (SalUCO, n = 7)

Creatine/saline infusions for all groups continued until postmortem. The infusion rate for all groups was set at 0.7 ml/h. Direct fetal infusion was necessary as creatine does not cross the sheep placenta,^20^ but is likely to cross the human placenta.^21^ Creatine infusion was prepared, as previously mentioned, using a dosage rate which has been shown to significantly increase cerebral creatine loading in fetal sheep.^7^ Every second day, an additional ~ 3 ml of fetal arterial blood was taken, centrifuged in ethylenediaminetetraacetic acid (EDTA) tubes for 10 minutes at 3,900 × *g*, 4°C to obtain plasma, then stored at −80°C for analysis of plasma creatine concentrations.

#### Fetal Umbilical Cord Occlusion

At 128 ± 2 dGA (~ 7 days after starting creatine/saline infusion), umbilical cord occlusion (UCO) was induced by inflating the umbilical cuff to an internal pressure of > 100 mmHg with 7 to 9 ml of sterile water to ensure complete cessation of umbilical blood flow. The researcher inducing the UCO was blinded to the treatment group. Successful occlusion was confirmed by rapid onset of fetal bradycardia (defined by an FHR < 100 bpm), and fetal hypoxemia and hypercapnia (confirmed by intra‐occlusion arterial blood gas assessment). Occlusion was terminated when MABP reached 19 mmHg. This blood pressure target was chosen because it enabled the induction of significant hypotension without leading to prolonged bradycardia or asystole after releasing the umbilical cord occluder. After deflating the umbilical cord occluder, the fetus was allowed to recover for 72 hours. Fetal arterial blood gases from the SalUCO and the CrUCO group were collected before UCO (−30 minutes), during UCO (5 minutes), immediately after release of the cuff (E; end), and then +1, +2, +4, +6, +24, +48, and +72 hours, respectively, relative to the start of UCO. Plasma was retrieved from the arterial samples collected at +1, +24, +48, and +72 hours, respectively.

#### Postmortem

Three days after UCO, the ewe and fetus were humanely euthanized with maternal intravenous injection of pentobarbitone sodium (~ 100 mg/kg; Lethabarb, Virbac Pty, Australia). At postmortem, the fetal brain was removed and weighed. The right cerebral hemisphere was immersion fixed in 4% paraformaldehyde (0.1 M; pH 7.4) for 72 hours and cut coronally into 5 mm‐thick rostro‐caudal blocks (~ 10 blocks per brain) and embedded in paraffin. The left cerebral hemisphere was flash frozen for future molecular analyses. Due to the global nature of the hypoxic insult, and based on previous research,^22^ there are no expected differences between the left and right hemispheres.

### Fetal Plasma Creatine Concentration Assay

Plasma samples were deproteinized with 1.5 M perchloric acid and neutralized with 2.1 M potassium hydrogen carbonate. Plasma creatine concentrations were determined using a fluorometric enzymatic assay, as previously described.^23^ The fluorescent signal was measured using a microplate reader (CLARIOStar Plus; BMG Labtech, Germany).

### Offline Analysis of Physiology Data

Offline analysis of physiological data was performed using LabChart Pro software. MABP, FHR, nuchal EMG, EEG Power, and SEF were processed as hourly averages and presented relative to the start of creatine infusion or UCO induction. For pre‐UCO and post‐UCO measurements, EEG power, SEF, and EMG activity are expressed as a change from baseline to account small intersubject variation within groups. The baseline for pre‐infusion data was calculated as the average measurement between −24 hours and up to 0 hours, when the saline/creatine infusion started. The baseline for pre‐ and post‐UCO data was calculated as the average measurement between −24 hours and up to 0 hours, when the UCO was induced. For intra‐occlusion analysis, physiological measurements were analyzed in 20‐second epochs and all intra‐occlusion physiology data are expressed as the change from the baseline average calculated from data obtained between −24 hours and up to 0 hours, when the UCO was induced. All physiological and seizure measurements were conducted on de‐identified recordings with assessors blinded to group allocation and any time‐specific indications. Analyses of physiological data post‐UCO were separated into 3 time‐blocks to represent the early recovery period (up to 6 hours post‐UCO), the intermediate recovery period (6 hours to 24 hours post‐UCO), and the late recovery period (24 hours to 72 hours post‐UCO).^7^

#### Seizures

Seizures were assessed during the 72 hours post occlusion recovery period. Seizures were identified as sudden, slow, repetitive, and evolving waveforms lasting longer than 10 seconds with an amplitude greater than 20 μV.^24^ Seizure burden was calculated as the total duration of seizure activity (minutes) during the recovery period. For temporal analysis of seizures, the total seizure duration (seconds) and number was averaged per hour. Seizure analysis was also conducted in EEG recordings from SalCon fetuses; however, no seizures were identified, and therefore seizure data are not reported for this group.

#### Assessment of Fetal Behavior

Assessment of fetal behavior was determined in hourly epochs from 24 hours prior to UCO to 72 hours post‐UCO. EEG and EMG activity were manually scored as either high or low amplitude in 10 minutes epochs over the 96 hours based on a voltage threshold calculated for each fetus (see Supplementary Materials and Methods and Baburamani et al^15^). In each 10‐minute epoch, fetal behavioral states were assessed as the incidence of nuchal EMG activity (fetal movement) occurring during high voltage (high amplitude) EEG activity (HVEEG + EMG) and incidence of low/reduced EMG activity (LEMG) occurring during low voltage EEG activity (LVEEG + LEMG). Abnormal fetal behavioral states were defined by the incidence of nuchal EMG activity occurring during LVEEG (LVEEG + EMG). All data were expressed as an hourly incidence (%), as described previously.^15^

#### Sleep‐State Cycling and EEG Background Activity

Grading of SSC was conducted in EEG segments that were 1 hour in length at relevant time points that included the baseline period (−24 hours), the beginning of major seizure activity (+6 hours), peak of seizure activity (+24 hours), and the end of the recovery period (+72 hours) post‐UCO, as described in Murray et al and Galinsky et al^1^, ^22^ Briefly, SSC was graded as normal (clearly defined periods of high and low frequency activity with each phase lasting ~ 10–20 minutes), poor (some state changes but not clearly defined), or absent (no SSC present). In the same 1‐hour epochs, background EEG activity was graded from 0 to 4 (as described in Murray et al^1^ and Supplementary Table S1); briefly: normal (0; continuous), mild abnormalities (1; continuous with abnormal activity), moderate abnormalities (2; discontinuous), major abnormalities (3; discontinuous with attenuated background patterns and/or interburst intervals), or inactive (4; isoelectric). EEG segments were deidentified from group and time point allocation to ensure blinding and randomization of qualitative assessment.

### Immunohistochemistry Analysis

Brain tissue sections were prepared as previously described in Tran et al^10^ and in Supplementary Materials and Methods and Supplementary Table S2. Sections (8 μm thick) were mounted onto superfrost slides and stained with anti‐neuronal nuclei (NeuN) for mature neurons; anti‐glial fibrillary acidic protein (GFAP) for astrocytes; and anti‐ionized calcium binding adaptor molecule 1 (IBA‐1) for microglia. An additional immunohistochemistry stain for cell death using a “terminal deoxynucleotidyl transferase dUTP nick end labeling” (TUNEL) assay (ApopTag Peroxidase In Situ Apoptosis Detection Kit, Millipore, USA) was conducted according to the manufacturer's instructions. Slides were scanned at 40× magnification using Aperio Scanscope AT Turbo (Leica Biosystems, Germany). Fields of view (410 μm × 320 μm) were analyzed in regions of interest that were selected based on an initial analysis of regional injury within the cortical and deep gray matter regions that are susceptible to hypoxic ischemic injury (Fig 1B). The rostral section (23 mm anterior to stereotaxic zero; frontal lobe) included the first and second parasagittal cortical gray matter (cortex layer III–IV) and intragyral and periventricular white matter (IGWM and PVWM, respectively). The caudal section (18 mm anterior to stereotaxic zero; parietal lobe) included the cortex, IGWM, PVWM, the dorsal hippocampal regions (cornu ammonis [CA] 1–4 regions and dentate gyrus [DG]). All analyses were undertaken by investigators (authors N.T.T. and J.S.) blinded to the treatment groups, and is described in detail in Supplementary Materials and Methods.

### Statistical Analysis

All data sets were analyzed for normal distribution using the Shapiro–Wilk test and statistical tests conducted, as outlined below. For repeated measurement analysis (physiological measurements, blood gas and chemistry, plasma creatine concentrations, SSC, and EEG background grading), 2‐way analysis of variance (ANOVA) tests were used to assess the interaction (*P*_Interaction_) and main effects of group (*P*_Group_) and time (*P*_Time_). Significant interactions and group effect were followed up with Tukey's multiple comparisons test. For comparison of seizure characteristics between the SalUCO and CrUCO groups, data were analyzed using a 2‐tailed, unpaired Student *t* test for parametric data or a Mann–Whitney test for nonparametric data. Immunohistochemistry and fetal characteristics were analyzed using a 1‐way ANOVA for parametric data or a Kruskal‐Wallis test for nonparametric data followed by a post hoc test using Tukey's multiple comparisons or Dunn's multiple comparisons, respectively. Incidence of seizures, SSC, and EEG background grade were analyzed using a 2‐sided chi‐square test. Statistical analysis and graphical data generation was conducted using GraphPad Prism version 8.0.0 (GraphPad Software, San Diego, CA, USA). All data are presented as mean ± standard deviation (SD). The value of *p* < 0.05 was considered statistically significant for all analyses.

## Results

### Creatine Supplementation Does Not Affect Basal Fetal Physiology

**FIGURE 2:**
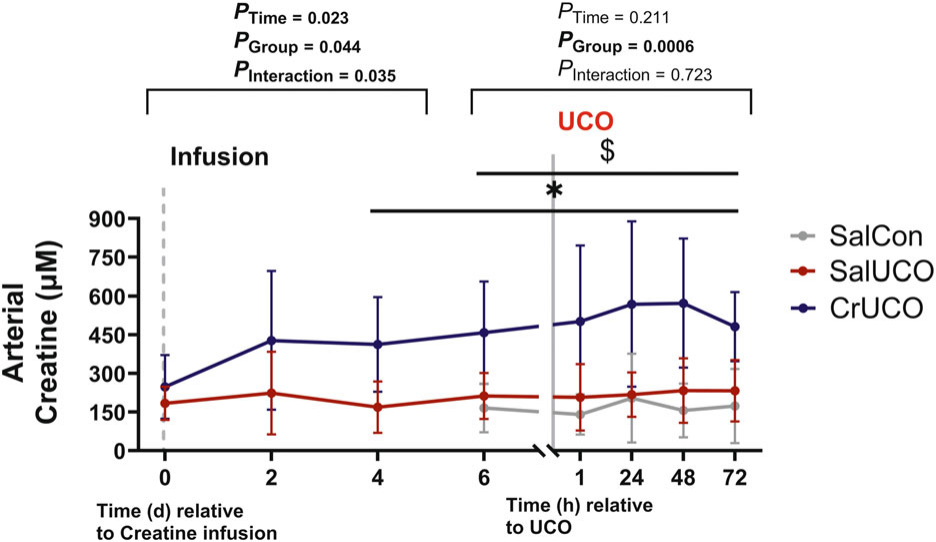
Arterial creatine concentrations increase with continuous creatine infusion. Data are mean ± SD. Two‐way repeated measures ANOVA; Tukey's post hoc test. $ and * denotes differences (*p* < 0.05) between SalCon and CrUCO, and SalUCO and CrUCO, respectively. SalCon, gray, n = 7; SalUCO, red, n = 7; CrUCO, blue, n = 7. ANOVA = analysis of variance; CrUCO = creatine umbilical cord occlusion; SalCon = saline infusion without umbilical cord occlusion; SalUCO = saline umbilical cord occlusion; SD = standard deviation. [Color figure can be viewed at www.annalsofneurology.org]

Creatine was continuously infused into the fetus at 121 dGA. Creatine infusion significantly increased arterial plasma creatine concentration after 4 days of infusion compared with SalUCO fetuses (*p* < 0.05; Fig 2). The higher level of plasma creatine in CrUCO fetuses compared with SalCon and SalUCO fetuses was maintained for the duration of the experiment. Creatine supplementation did not alter fetal cardiovascular or neural physiology or blood gas and chemistry (Supplementary Figs S2 and S3). All fetuses were of similar body and brain weight at postmortem (Supplementary Table S3). Baseline (−24 hours up to 0 hours, when the UCO was induced) cardiovascular and neurophysiological parameters were not different between groups (*P*_*Group*_ and *P*_*Interaction*_ >0.1; Supplementary Table S4).

### Effect of Creatine Supplementation on Systemic Physiological Responses to Acute UCO

**FIGURE 3:**
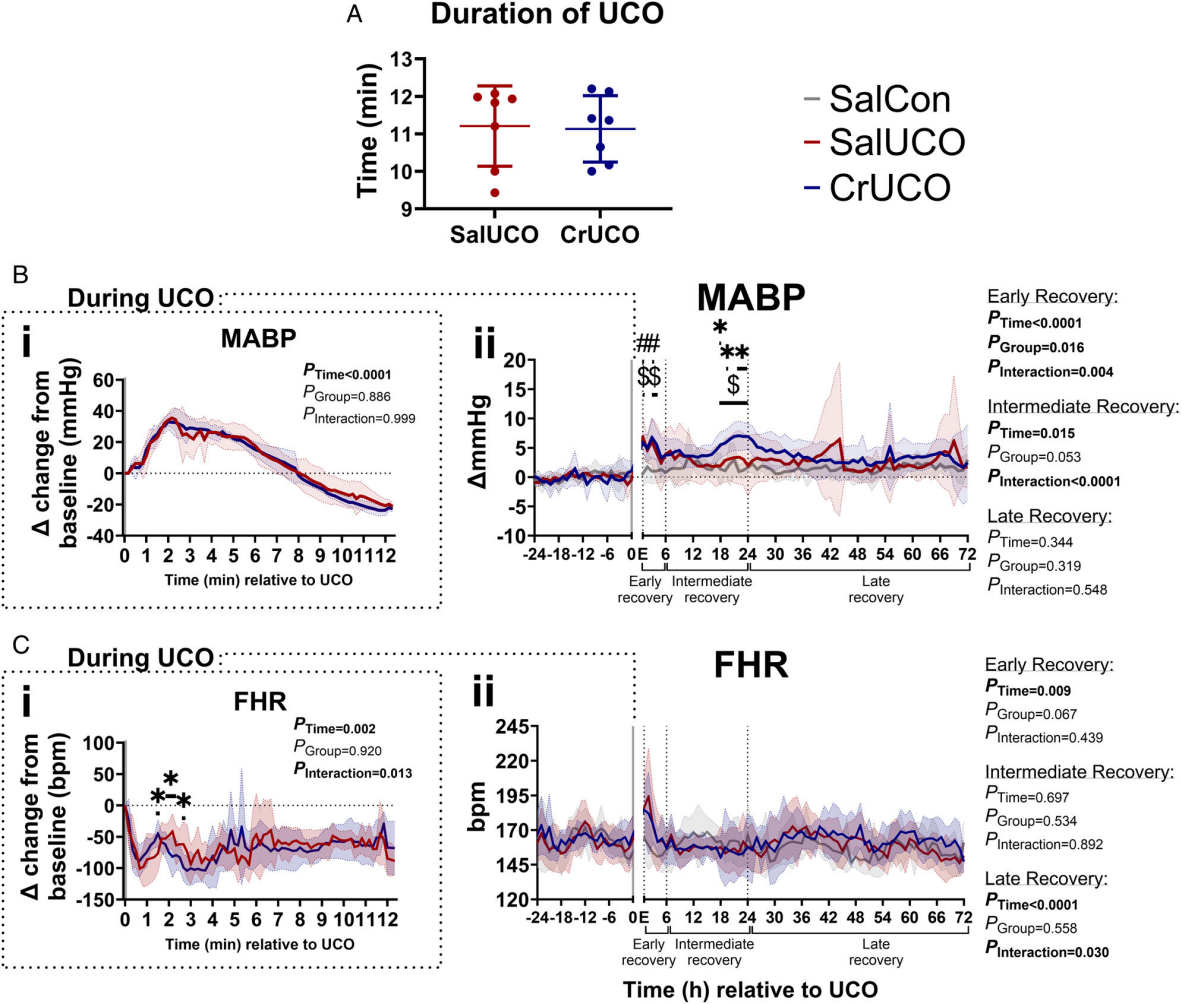
Prophylactic creatine supplementation alters the systemic physiological response immediately upon UCO induction and after UCO. (A) The duration of complete UCO until MABP reached 19 mmHg in SalUCO and CrUCO fetuses. Systemic physiological recordings of (B) MABP and (C) FHR. (i) Dashed boxes are measurements during UCO; data are 20‐second epochs and presented as delta (Δ) change from baseline. (ii) Data analysis during early (0–6 hours), intermediate (6–24 hours), and late (24–72 hours) recovery periods are 1‐hour epochs. MABP is presented as Δ change from baseline. All data are presented as mean ± SD. The *x‐*axis shows time relative to the UCO, which is indicated by a gray bar; E indicates end of UCO. Two‐way repeated measures ANOVA with Tukey's post hoc test. *#* shows significant differences (*p* < 0.05) between SalCon and SalUCO, $ = significant differences (*p* < 0.05) between SalCon and CrUCO, * = significant differences (*p* < 0.05) between SalUCO and CrUCO. SalCon, gray, n = 7; SalUCO, red, n = 7; CrUCO, blue, n = 7. ANOVA = analysis of variance; CrUCO = creatine umbilical cord occlusion; FHR = fetal heart rate; MABP = mean arterial blood pressure; SalCon = saline infusion without umbilical cord occlusion; SalUCO = saline umbilical cord occlusion; SD = standard deviation; UCO = umbilical cord occlusion. [Color figure can be viewed at www.annalsofneurology.org]

To simulate moderate acute asphyxia, fetuses at 128 ± 2 dGA were subjected to complete UCO until the MABP fell to 19 mmHg. Creatine pretreatment did not affect the time taken for the MABP to reach 19 mmHg (*p* = 0.932; Fig 3A). The average MABP nadir was 19.86 ± 1.50 mmHg for both groups and was not statistically different (*p* = 0.805). UCO was characterized by a transient increase in the MABP and sustained bradycardia followed by a reduction in the MABP below baseline from approximately 8.17 minutes after inducing UCO (Fig 3B[i], C[i]). The transient increase in the FHR during bradycardia occurred earlier in CrUCO fetuses compared with SalUCO (*p* < 0.04). By 3 minutes into the UCO, the FHR was similar between CrUCO and SalUCO fetuses.

Following UCO, the 72 hours of recovery was divided into an early (0–6 hours), intermediate (6–24 hours), and late (24–72 hours) recovery period. After deflating the umbilical cord occluder and reperfusion of umbilical blood flow, the MABP and FHR increased rapidly in both UCO groups at 1 hour and again at 3 hours with the secondary peak increase in the MABP occurring for a further hour in CrUCO fetuses, but not in SalUCO fetuses (Fig 3B[ii]). During the intermediate recovery period (6–24 hours), there was a small secondary increase (~ 2–3 mmHg) in the MABP in CrUCO fetuses compared with the SalUCO and SalCon groups between 18 and 24 hours post‐UCO. Thereafter, the MABP did not differ between the groups. No differences between groups were found for the FHR during the recovery period (see Fig 3C[ii]).

Immediately after UCO, CrUCO fetuses had higher levels of PaO_2_ (SalUCO = 13.71 ± 2.41 mmHg and CrUCO = 16.36 ± 1.62 mmHg, *p* = 0.050) and SaO_2_ (SalUCO = 17.87 ± 6.87% and CrUCO = 25.23 ± 6.30%, *p* = 0.025; Supplementary Fig S4) compared with the SalUCO group. The magnitude of acidosis (reduction in pH, hypercapnia, and hyperlactatemia) did not differ between the UCO groups. During early recovery, up to 6 hours post‐UCO, arterial pH was not significantly higher in CrUCO fetuses compared with SalUCO (*P*_GROUP_ = 0.053). By late recovery, arterial blood biochemistry did not differ between the UCO groups.

### Effect of Creatine Supplementation on Neurophysiological Responses During and After UCO

**FIGURE 4:**
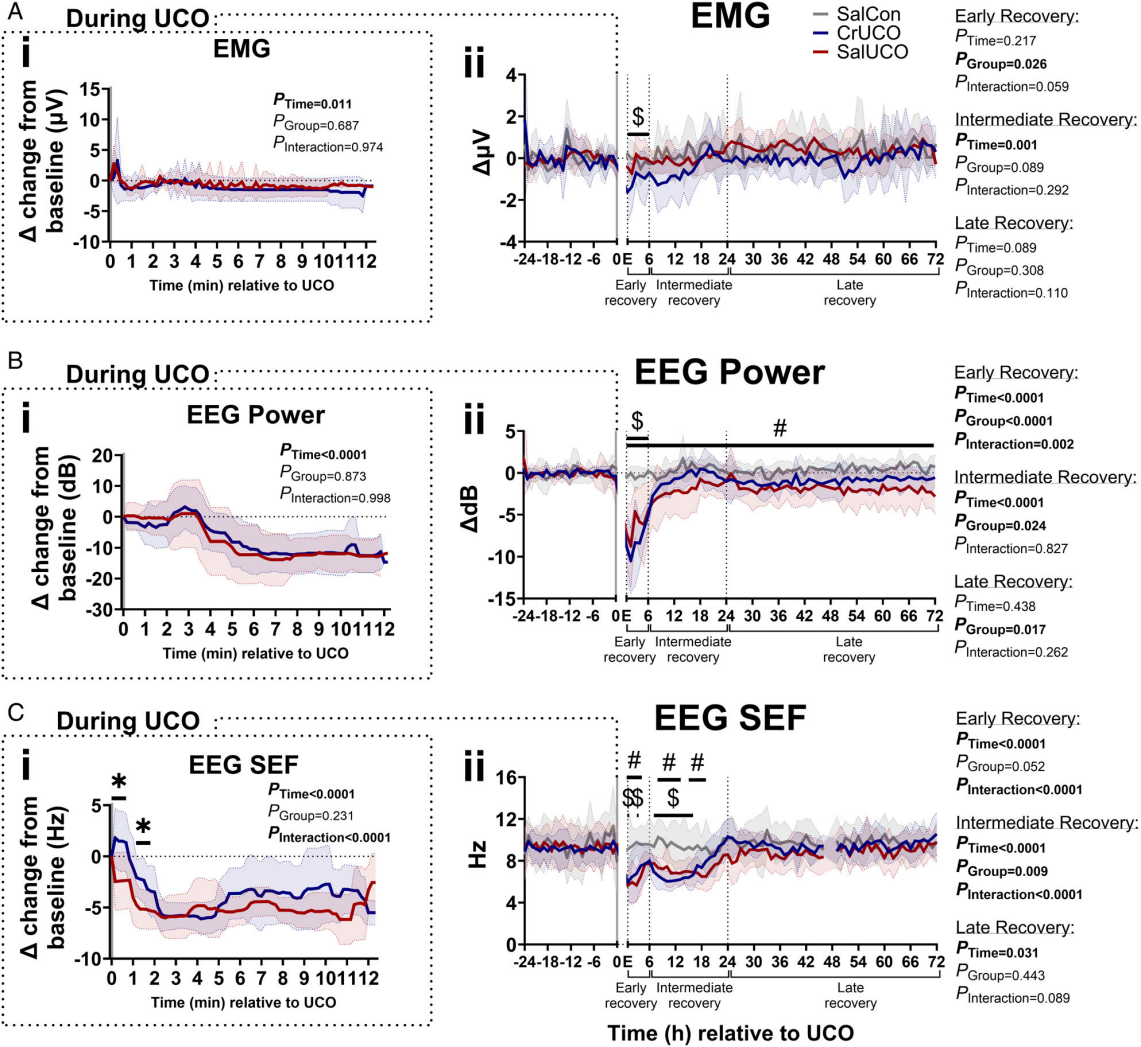
Prophylactic creatine supplementation alters the neurophysiological response immediately upon UCO induction and after UCO. Neurophysiological changes over time. From the top down, the figure shows (A) nuchal EMG and EEG (B) power, and (C) SEF. (i) Dashed boxes are measurements during UCO, data are 20‐second epochs and presented as delta (Δ) change from baseline. (ii) Data analysis during early (0–6 hours), intermediate (6–24 hours), and late (24–72 hours) recovery periods are 1‐hour epochs. EMG and EEG power are presented as Δ change from baseline. All data are presented as mean ± SD. The *x‐*axis shows time relative to the UCO, which is indicated by a gray bar; E indicates end of UCO. Two‐way repeated measures ANOVA, Tukey's post hoc test. *#* = significant differences (*p* < 0.05) between SalCon and SalUCO; $ = significant differences (*p* < 0.05) between SalCon and CrUCO; * = significant differences (*p* < 0.05) between SalUCO and CrUCO. SalCon, gray, n = 7; SalUCO, red, n = 7; CrUCO, blue, n = 7. ANOVA = analysis of variance; CrUCO = creatine umbilical cord occlusion; EEG = electroencephalogram; EMG = electromyography; SalCon = saline infusion without umbilical cord occlusion; SalUCO = saline umbilical cord occlusion; SD = standard deviation; SEF = spectral edge frequency; UCO = umbilical cord occlusion. [Color figure can be viewed at www.annalsofneurology.org]

The effects of creatine pretreatment on the immediate neurophysiological adaptation to UCO and reperfusion were assessed from EEG and EMG recordings (Fig 4 and Supplementary Fig S5). The induction of UCO caused similar suppression of nuchal EMG activity and EEG power in both groups (Fig 4A[i], B[i]). During the first 2.5 minutes of UCO, suppression of total EEG frequency was slower in the CrUCO fetuses compared with the SalUCO group, with an associated higher proportion of activity in the theta, alpha and beta frequency‐bands compared with the SalUCO fetuses (Fig 4C[i] and Supplementary Fig S5A–D[i]). For the remainder of the UCO period, EEG frequency did not differ between the occlusion groups.

After UCO, nuchal EMG activity in the CrUCO fetuses remained decreased during the entire early recovery period (first 6 hours after UCO) compared with both the SalCon and SalUCO fetuses (*p* = 0.041 and 0.051, respectively; see Fig 4A[ii]). Thereafter, nuchal EMG activity in the CrUCO group did not differ from the SalCon and SalUCO groups.

EEG power remained suppressed immediately after UCO in both groups (see Fig 4B[ii]). CrUCO fetuses had suppression of EEG power compared with SalCon fetuses only during the first 6 hours of recovery (*p* < 0.05), and returning to near SalCon levels between 6 and 72 hours of recovery. In the SalUCO group, EEG power remained lower compared with SalCon fetuses throughout the entire 72 hours of recovery (*p* < 0.02).

After UCO, the SEF remained decreased in the SalUCO group up to 4 hours after UCO, but only at 1 and 3 hours after UCO in CrUCO fetuses compared with SalCon fetuses (see Fig 4C[ii]). A secondary transient decrease in EEG SEF occurred in both UCO groups during the intermediate recovery period. The recovery of EEG SEF to SalCon levels occurred earlier in CrUCO fetuses compared with SalUCO fetuses (17 hours vs 20 hours post‐UCO). There were no differences between SalUCO and CrUCO fetuses in the recovery of frequency bands after UCO (see Supplementary Fig S5A–D[ii]).

### Creatine Supplementation Alters the Coordination of EEG and Nuchal EMG Activity after UCO

**FIGURE 5:**
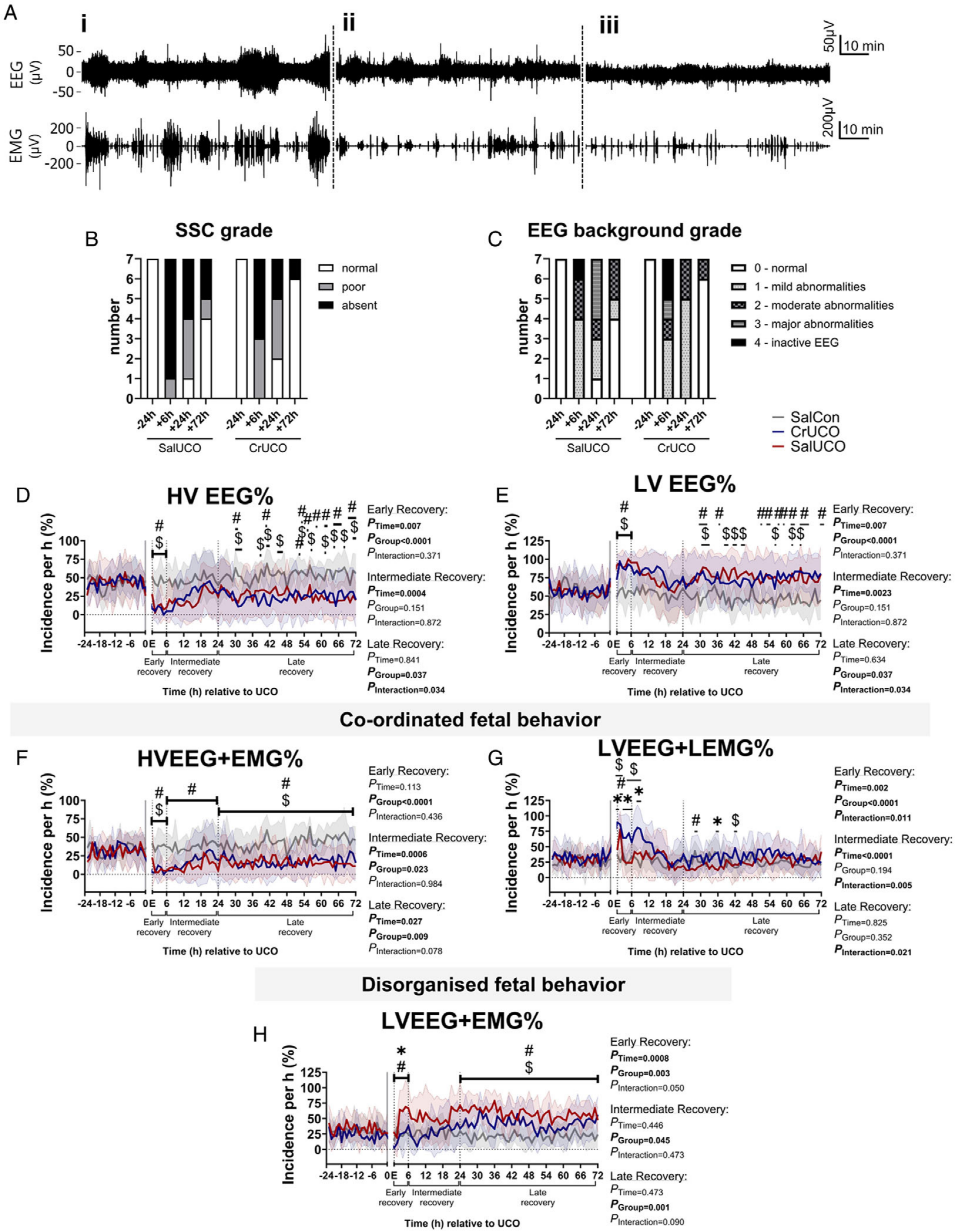
Creatine supplementation does not rescue long‐term disorganization of fetal behavior. (A)[i] Raw traces of normal cortical EEG and nuchal EMG activity representing alternating coordinated periods of HVEEG with EMG activity then periods of LVEEG and LEMG activity. Representative traces demonstrate [ii] poor and [iii] absent coordinated fetal behavioral states. (B) The number of fetuses with normal (white), poor (gray) and absent (black) SSC. (C) The number of fetuses with EEG background scores of 0 (normal) to 4 (isoelectric and inactive). Mann–Whitney tests. Incidence of (D) HVEEG, and (E) LVEEG, per hour during the baseline (−24 to 0 hours) and 72 hours of recovery. Incidence of coordinated fetal behavior, as shown by (F) HVEEG and EMG activity and (G) LVEEG and LEMG activity. Incidence of disorganized fetal behavior, as shown by (H) LVEEG and EMG activity per hour. All data are 1‐hour epochs and presented as mean ± SD. The *x‐*axis is time relative to UCO indicated by a gray bar; E indicates end of UCO. Two‐way repeated measures ANOVA, Tukey's post hoc test. *#* = significant differences (*p* < 0.05) between SalCon and SalUCO; $ = significant differences (*p* < 0.05) between SalCon and CrUCO; * = significant differences (*p* < 0.05) between SalUCO and CrUCO. SalCon, gray, n = 7; SalUCO, red, n = 7; CrUCO, blue, n = 7. ANOVA = analysis of variance; CrUCO = creatine umbilical cord occlusion; EEG = electroencephalogram; EMG = electromyography; HVEEG = high voltage electroencephalogram; LEMG = low electromyography; LVEEG = low voltage electroencephalogram; SalCon = saline infusion without umbilical cord occlusion; SalUCO = saline umbilical cord occlusion; SD = standard deviation; SEF = spectral edge frequency; SSC = sleep state cycling; UCO = umbilical cord occlusion. [Color figure can be viewed at www.annalsofneurology.org]

Late gestation fetal sheep develop steady SSC described as episodes of alternating periods of high amplitude/voltage and low amplitude/voltage EEG with coordinated periods of increased nuchal EMG activity and low EMG activity or atonia, respectively (Fig 5A). SSC or the coordination of EEG and EMG reflects the normal development of functional brain networks and their organization and was present by ~ 126 dGA.^25^ Prior to UCO, SSC and normal EEG background activity was observed in all fetuses (Fig 5B, C). Before UCO the incidence of high voltage EEG and low voltage EEG per hour were similar between groups (Fig 5D, E; and see Supplementary Table S4), as was the incidence of coordinated HVEEG with increased nuchal EMG activity (HVEEG + EMG) and LVEEG with reduced EMG activity (LVEEG + LEMG; Fig 5F, G, and see Supplementary Table S4). Before UCO, the incidence of disorganized fetal behavior, as measured by the incidence of low voltage EEG periods occurring with increased nuchal EMG activity (LVEEG + EMG) was similar between groups (Fig 5H, and see Supplementary Table S4).

After UCO, the incidence of HVEEG decreased, and LVEEG increased in both the SalUCO and CrUCO fetuses during the early recovery period (0–6 hours) compared with SalCon (see Fig 5D, E), consistent with suppression of EEG power after UCO (Fig 4B[ii]). The grades of the EEG background activity were not different between the occlusion groups at 6, 24, or 72 hours post‐UCO, with no significant difference in the number of fetuses that had normal EEG background activity in the CrUCO group (n = 6/7) compared with the SalUCO group (n = 4/7) by the end of the 72‐hour recovery period (*χ* ^2^ = 1.40, *p* = 0.237; see Fig 5C).

The incidence of coordinated HVEEG + EMG was reduced throughout the entire 72 hours after UCO in the SalUCO fetuses (*P*_*EarlyRecovery*_ <0.0001, *P*_*IntermediateRecovery*_ = 0.023, and *P*_*LateRecovery*_ = 0.016 vs SalCon), but was only reduced during the early and late recovery period in CrUCO fetuses (*P*_*EarlyRecovery*_ < 0.0001, *P*_*LateRecovery*_ = 0.019 vs SalCon; see Fig 5F). The incidence of LVEEG + LEMG increased after UCO in the CrUCO fetuses and was higher compared with both the SalCon and SalUCO fetuses up to 9 hours after UCO (*p* < 0.05; see Fig 5G). The incidence of LVEEG + LEMG was higher in SalUCO compared with SalCon fetuses only at 2 hours after UCO. After 9 hours, the incidence of LVEEG + LEMG was similar between groups.

The development of disorganized fetal behavior during the recovery period was measured by quantifying the incidence of LVEEG during EMG activity and poor/absent SSC (see Fig 5B, H). In the SalUCO fetuses, disorganized fetal behavior was evident by increased LVEEG + EMG during the early recovery period (*p* = 0.012 vs SalCon, *p* = 0.004 vs CrUCO; see Fig 5H). At the end of the early recovery period (6 hours), SSC was absent in 6 of 7 SalUCO fetuses compared with 3 of 7 CrUCO fetuses (see Fig 5B). During the late recovery period, both the SalUCO and CrUCO fetuses had increased incidence of LVEEG + EMG compared with SalCon (*p* = 0.0008 and *p* = 0.042, respectively; see Fig 5H). At 72 hours post‐UCO, SSC had returned in 4 of 7 SalUCO and 6 of 7 CrUCO fetuses (*χ* ^2^ = 1.40, *p* = 0.237; see Fig 5B).

### Creatine Supplementation Reduces Electrographic Seizure Burden

**FIGURE 6:**
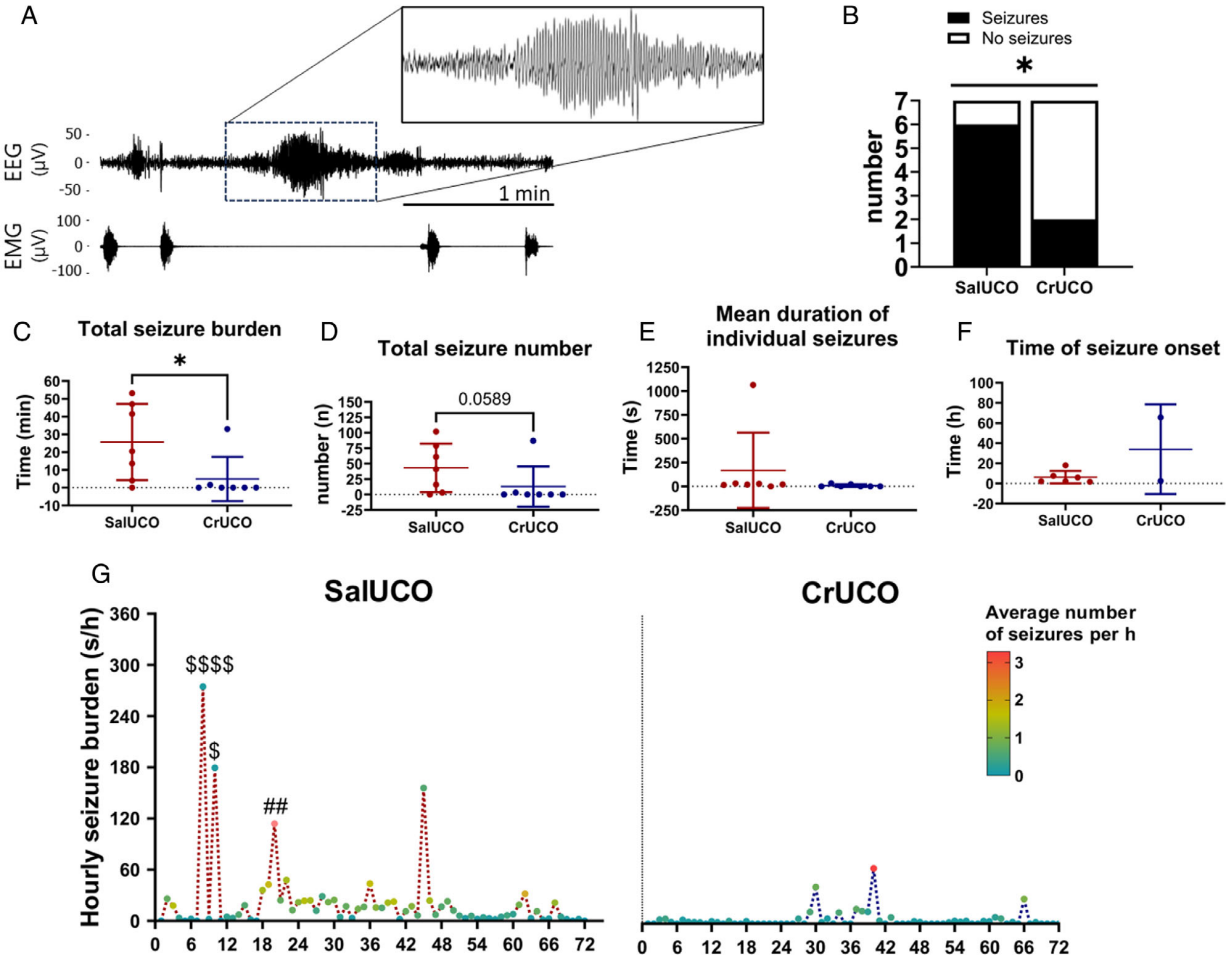
Prophylactic creatine supplementation reduces seizure burden. (A) Representative EEG showing a single seizure event. Note the absence of nuchal EMG which is a key seizure characteristic. (B) Incidence of seizures in SalUCO and CrUCO groups. (C) Total seizure burden, (D) total seizure number, (E) duration of individual seizures, and (F) time to seizure onset. All data are mean ± SD. Mann–Whitney test. Significant differences between SalUCO and CrUCO indicated by *p < 0.05. (G) Temporal analysis of seizures showing hourly seizure burden and numbers of seizures. The *Y*‐axis indicates average seizure burden in seconds (s)/hour in SalUCO, n = 7 and CrUCO, n = 7, the *X‐*axis shows time relative to UCO, and the color scale indicates average number of seizures/hour. Two‐way repeated measures ANOVA, Tukey's post hoc test. $ = significant differences (*p* < 0.05) in seizure duration; # = significant differences (*p* < 0.05) in seizure number between SalUCO and CrUCO groups. ANOVA = analysis of variance; CrUCO = creatine umbilical cord occlusion; EEG = electroencephalogram; EMG = electromyography; SalUCO = saline umbilical cord occlusion; SD = standard deviation; UCO = umbilical cord occlusion. [Color figure can be viewed at www.annalsofneurology.org]

Seizures occurred in 6 of 7 SalUCO fetuses and only 2 of 7 CrUCO fetuses (*χ* ^2^ = 1.67, *p* = 0.031; Fig 6A, B). Total seizure burden was significantly reduced in the CrUCO fetuses compared with SalUCO (*U* = 7.5, *p* = 0.026; Fig 6C). The total number of seizures was not significantly reduced in the CrUCO fetuses compared with the SalUCO fetuses (*U* = 10, *p* = 0.059; Fig 6D). The mean duration of individual seizures did not differ between groups (Fig 6E). The time of seizure onset was between 1.55 and 17.95 hours in the SalUCO fetuses. In the 2 CrUCO fetuses that presented with seizures, seizure onset was at 2.48 and 65.53 hours post‐UCO (Fig 6F). Temporal analysis of electrographic seizures indicated that most of the seizure activity occurred in the SalUCO fetuses between 6 and 48 hours post‐UCO. In the SalUCO fetuses, the average seizure duration was higher at 8 and 10 hours post‐UCO and the average number of seizures was higher at 20 hours post‐UCO, compared with CrUCO fetuses (*p* < 0.05; Fig 6G).

### Creatine Supplementation Reduces Cell Death and Numbers of Astrocytes but Does Not Affect Numbers of Microglia After UCO

**FIGURE 7:**
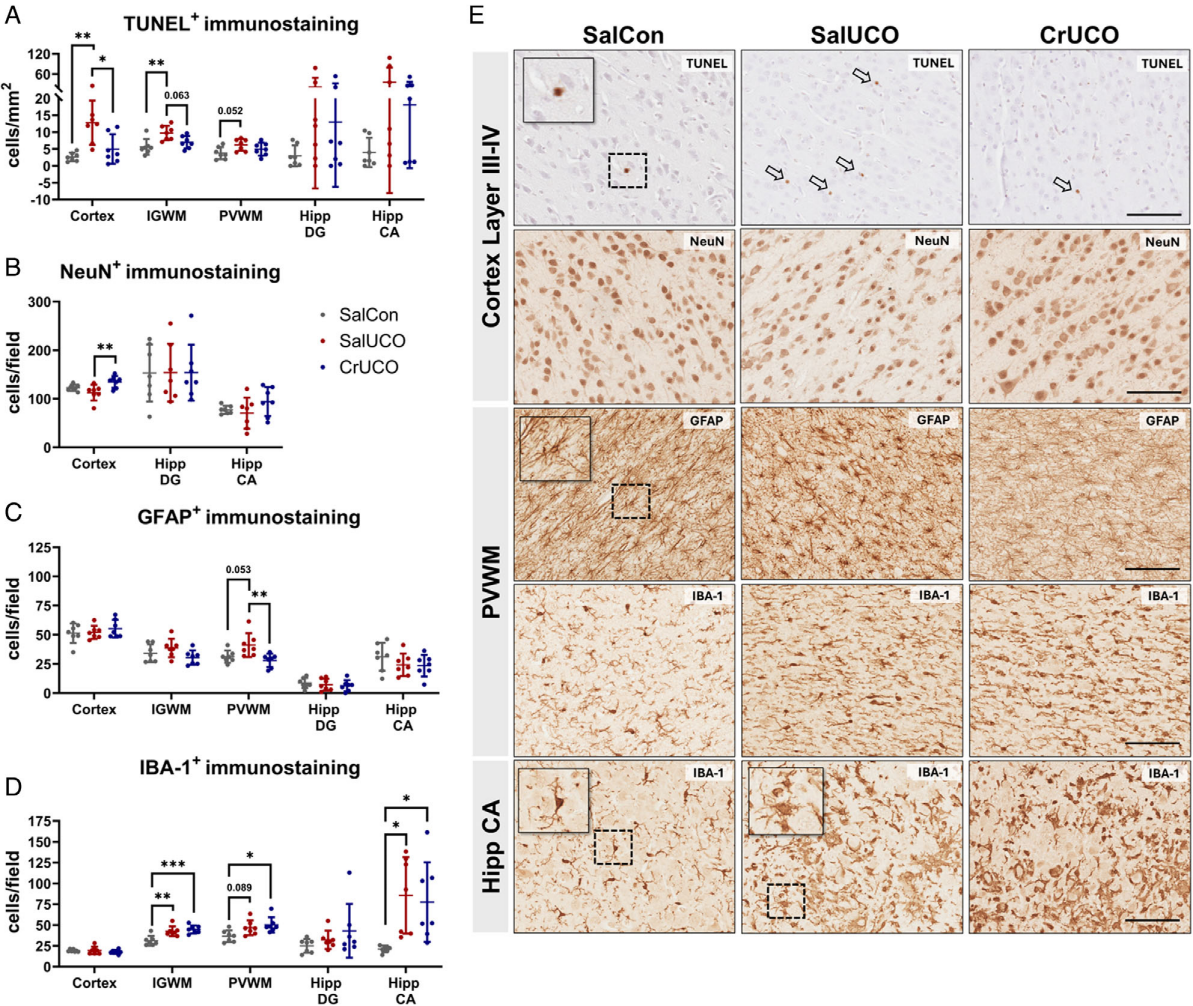
Prophylactic creatine supplementation ameliorates histological pathology following UCO. Numbers of (A) TUNEL^+^ cells, (B) NeuN^+^, (C) GFAP^+^ astrocytes, and (D) IBA‐1^+^ microglia in the cortical grey matter (cortex), IGWM, PVWM, Hipp DG, and CA region. (E) Representative images at 20× magnification. Scale bars are 100 μm. All data are mean ± SD. SalCon, n = 7; SalUCO, n = 7; CrUCO, n = 7. One‐way ANOVA with Tukey's or Kruskal‐Wallis with Dunn's post hoc comparisons. *p < 0.05, **p < 0.01, **p < 0.001. ANOVA = analysis of variance; CA = cornu ammonis; CrUCO = creatine umbilical cord occlusion; DG = dentate gyrus; GFAP^+^ = glial fibrillary acidic protein; Hipp = hippocampus; IBA‐1^+^ = ionized calcium binding adaptor molecule 1; IGWM = intragyral white matter; NeuN^+^ = neuronal nuclei; PVWM = periventricular white matter; SalCon = saline infusion without umbilical cord occlusion; SalUCO = saline umbilical cord occlusion; TUNEL^+^ = terminal deoxynucleotidyl transferase dUTP nick‐end labeling; UCO = umbilical cord occlusion.

Numbers of TUNEL^+^ cells, a marker of cell death, were higher in SalUCO fetuses compared with SalCon in the cerebral cortex (*p* = 0.002) and IGWM (*p* = 0.005; Fig 7A, E). In the cerebral cortex of the CrUCO fetuses, numbers of TUNEL^+^ cells were significantly reduced compared with SalUCO (*p* = 0.014). Numbers of TUNEL^+^ cells in the IGWM of CrUCO fetuses were not significantly lower than SalUCO (*p* = 0.063). In the PVWM, compared with SalCon, numbers of TUNEL^+^ cells were not significantly increased in the SalUCO group (*p* = 0.052), and did not differ from CrUCO fetuses (*p* = 0.460). In the cerebral cortex, the number of NeuN^+^ cells were higher in the CrUCO fetuses compared with the SalUCO group (*p* = 0.009; Fig 7B, E).

Numbers of GFAP^+^ astrocytes in the PVWM were higher in SalUCO fetuses compared with SalCon, but this did not reach statistical significance (*p* = 0.053; Fig 7C, E). In the PVWM, the numbers of GFAP^+^ astrocytes were significantly lower in the CrUCO fetuses compared with SalUCO (*p* = 0.010).

Numbers of IBA‐1^+^ microglia were increased in SalUCO and CrUCO groups compared with SalCon in the IGWM (*p* = 0.003 and 0.0006, respectively), PVWM (*p* = 0.089 and 0.019, respectively) and the CA of the hippocampus (*p* = 0.015 and 0.034, respectively; Fig 7D, E).

## Discussion

This study found that creatine supplementation before and after HI in late gestation fetal sheep improved the recovery of EEG power, reduced the overall burden of electrographic seizures, and mitigated regional cerebral cell death and astrocytosis. In addition, the data showed that the 6 to 7‐day baseline period of fetal creatine infusion (before UCO) did not alter the physiological status of the fetus, further supporting its safety profile. Overall, these results demonstrate that fetal creatine supplementation is protective against acute asphyxia in late pregnancy and thus presents a possible neuroprotective strategy for perinatal HIE.

### Creatine Pretreatment Reduces Post‐Asphyxia Seizure Burden

The major novel finding of this study was that prophylactic creatine significantly reduced seizure burden after UCO. The anticonvulsant properties of creatine were distinct during the intermediate recovery period (6–24 hours), a key period of secondary energy failure following HIE. The mechanisms by which creatine reduces hypoxia‐induced seizures could be direct or indirect. Direct mechanisms might include neuromodulation to influence cortical excitability^12^, ^13^ and/or NMDA receptor activation.^26^ The indirect mechanisms might include delaying anoxic depolarization,^27^ neuronal Na^+^/K^+^‐ATPase activity modulation,^28^ and/or protection of neuronal integrity given the mitigated cell death and astrogliosis observed within the cortex and major white matter tracts. Time‐dependent metabolic alterations, as evidenced by the multiple systemic and central physiological alterations both at the onset and after the brief period of acute *in utero* asphyxia in creatine treated fetuses, may have also reduced cerebral metabolic and oxidative stress responses^8^ contributing to the creatine‐mediated reduced seizure burden. A prospective cohort study conducted by Glass et al, strongly suggests that seizures can themselves cause brain damage, due to the association between neonatal seizures and neurodevelopmental outcomes of term infants independent of the severity of the HI insult.^17^ The reduction in electrographic seizures conferred by creatine even by 72 hours is likely associated with an accompanying reduction in the risk of long‐term adverse neurological outcomes.

### Creatine Pretreatment Improves the Central and Systemic Physiological Responses to Asphyxia

At the onset of asphyxic hypoxia, the immediate response is initial suppression of EEG. This is an adaptative response driven by active suppression by inhibitory neuromodulators, such as adenosine as a means to extend residual anaerobic reserves to prevent anoxic depolarization.^29^ We found that within the first 2 minutes of UCO, creatine‐supplemented fetuses responded with a delayed suppression of EEG frequency with higher proportions of activity in the theta, alpha, and beta frequency‐bands compared with SalUCO fetuses. The increased cerebral energy reserves provided by creatine supplementation could have delayed anoxic depolarization, allowing for better maintenance of cerebral electrical activity, as reported previously in guinea pig hippocampal slices.^11^ Following these central physiological changes from UCO, the peripheral chemoreflex induces an autonomically driven increase in MABP and bradycardia. Within the next minute, a unique subsequent physiological response of near‐term fetuses is a brief transient increase in FHR during the early phase of complete umbilical cord occlusion, a response thought to be due to increased sympathetic activity and circulating catecholamines,^30^, ^31^ and/or a reduction in parasympathetic drive.^32^ This brief FHR adaptation occurred earlier in creatine‐supplemented fetuses, which may be explained by a faster attenuation of parasympathetic activity,^33^ or a vasodilatory effect of creatine^34^ that increases blood flow to the adrenal medulla to increase circulating catecholamines. Despite these early differences, by 3 minutes of UCO, the physiological responses were similar in both occlusion groups. Creatine did not improve the ability of the fetus to sustain a longer duration of UCO until MABP reached 19 mmHg. This is likely because blood pressure is eventually determined by a combination of hypoxia‐induced vascular relaxation and reduced cardiac output rather than autonomic reflex responses.^32^

At reinstatement of umbilical blood flow after UCO, there were differences in how creatine‐supplemented fetuses responded during the early, intermediate, and later recovery periods. Immediately at reperfusion, the arterial SaO_2_ and PaO_2_ were higher in creatine‐supplemented fetuses, potentially reflecting a more rapid recovery of arterial blood oxygen levels. We did not measure cerebral blood flow or cerebral oxygen metabolism; however, blood sampling from the right brachial artery provides insight into the approximate arterial oxygenation in the right carotid artery. Higher arterial SaO_2_ and PaO_2_ suggest that creatine‐supplemented fetuses had improved cerebral oxygen delivery. Improved cerebral oxygenation immediately after UCO may explain the improved neurophysiological responses after umbilical blood flow reperfusion.

During the early recovery period (0–6 hours post‐UCO), creatine fetuses did not display disorganized fetal behavior but did have reduced nuchal EMG activity. This was followed by faster recovery of EEG power and frequency during the intermediate and late recovery periods (> 6 hours post‐UCO). Morever, by 72 hours creatine‐treated fetuses had reduced numbers of TUNEL^+^ cells in the cerebral cortex (suggesting a reduction in cell death) and a trend (*p*=0.063) for reduced numbers of TUNEL^+^ cells in the subcortical (intragyral) white matter compared with saline treated fetuses. Despite observing increased numbers of cortical neurons in creatine fetuses compared with saline fetuses after UCO, we did not observe a concomitant reduction in numbers of cortical neurons in the saline UCO group compared with saline controls. Thus, we cannot conclude that the increased cell death observed as a result of the UCO was solely attributed to increased neuronal death. Moreover, creatine fetuses had reduced numbers of astrocytes in periventricular white matter compared with saline fetuses after UCO, suggesting a reduction in astrocytosis.

Improved provision of high‐energy phosphates in the brain afforded by creatine supplementation has been associated with mitigation of cerebral and brainstem ATP depletion,^35^, ^36^ improved oxidative metabolism,^37^ and reductions in cerebral reactive oxygen species and lactate accumulation after HI injury^8^—effects overall that could be expected to improve EEG activity and reduce brain cell death. Astrocytes also rely on the creatine energy buffering pathway to maintain cellular ATP, especially during impaired glycolysis and mitochondrial oxidative phosphorylation.^38^ Although the exact mechanism/s underpinning the reduction in astrocytosis in the large white matter tracts of creatine‐treated fetuses was not investigated, we may reasonably speculate this was attributed to a reduction in seizures in the creatine group. Indeed, seizures have been shown to trigger astrocytosis by promoting the extracellular release of excitatory amino acids, ATP, and opening of glial/neuronal hemichannels.^39^ Therefore, the anti‐excitotoxicity effect of creatine observed after UCO most likely explains the reduction in white matter astrocytosis in the CrUCO group.

Creatine treatment did not affect numbers of microglia (IBA^+^ staining) in the subcortical white matter regions and hippocampus induced by the UCO, despite the reduced cell death and astrogliosis. Indeed, both occlusion groups showed a similar increase in numbers of microglia compared with saline controls. These data indicate that creatine mediated neuroprotection is unlikely to involve direct anti‐inflammatory effects and are consistent with data reported in an *in vitro* model of demyelinating injury.^40^ Thus, creatine is unlikely to completely ameliorate HIE pathology, specifically tissue damage involving inflammatory pathways. Regardless, reduced seizure burden, improved EEG recovery, and reduced cerebral pathology resulting from fetal creatine infusion are significant clinical outcomes.

Whilst mitigation of neurophysiological pathology is one possible interpretation of our results, it is also plausible that creatine‐supplementation afforded a delay in the manifestations of pathology; that is, delayed the onset of the characteristic secondary energy failure that occurs after HI injury. This is supported by the eventual increase in disorganized fetal behavior observed in the late recovery stage (> 24 hours post‐UCO) in creatine‐treated fetuses, as indicated by the increased incidence of EMG activity occurring during low‐voltage EEG and the delayed seizure onset of one of the 2 CrUCO fetuses that presented with seizures.

### Clinical Implications of a Prophylactic Creatine Treatment for HIE

The pathophysiological outcomes observed after UCO in this study were consistent with mild to moderate asphyxial encephalopathy; that is, pH > 7 and base excess >−12, which is a significant contributor to long‐term neurodevelopmental impairment.^41^ In addition, by 72 hours after UCO, the incidence of poor/absent SSC and abnormal EEG background in 3 of 7 saline fetuses indicate a varying degree of HIE was caused by the brief interruption of umbilical blood flow. In a clinical setting, difficulty in identifying pathology associated with HIE, the variability in presentation, as well as the lack of access to therapeutic hypothermia and continuous EEG monitoring in LMICs all combine to contribute to the current lack of effective treatment strategies that can be administered in a timely and effective manner globally.

Therefore, a prophylactic treatment, such as maternal creatine supplementation, during pregnancy for the human fetus could provide readily accessible protection for unpredictable HI insults. Not only is there now considerable safety, tolerance, and efficacy of creatine supplementation in preclinical models of perinatal HIE,^42^, ^43^, ^44^ significant associations of creatine metabolism perturbations with fetal, placental, newborn, and maternal outcomes in human pregnancy have been identified.^6^, ^45^, ^46^ Moreover, the marked reduction in electrographic seizure burden with creatine pretreatment up to 72 hours post‐UCO demonstrates real potential for improving neonatal outcomes. It may also help to address the current limitations of seizure management and identification.^47^ Whether these neuroprotective effects of creatine apply to more severe cases of hypoxic injury and/or as an adjunct therapy to therapeutic hypothermia is also worth investigating. Together, these preclinical results highlight that leveraging creatine metabolism via supplementation during pregnancy might provide a viable and safe solution to mitigate adverse newborn outcomes in conditions such as HIE.

Further studies to support prophylactic antenatal creatine supplementation during pregnancy for HIE and seizures are required to address limitations in this study. First, this study investigated histological outcomes from a single timepoint during the secondary phase of HIE. It is well established that the pathogenesis of HIE is an evolving process, therefore subsequent assessments of neuropathology beyond the secondary phase would improve our understanding of how creatine modulates longer‐term HIE pathophysiology. Second, the current study was not powered to specifically address sex dependent effects. Allocation to groups were randomized and blinded until postmortem, leading to a skewed male:female ratio in the CrUCO group (6:1). Indeed, studies in fetal sheep have indicated that males have more seizures after UCO,^48^ and in neonatal mice exposed to HI, males have increased cerebral injury compared with females 72 hours after recovery.^49^ Despite having more male subjects in the CrUCO cohort, we observed a reduction in seizure burden and modest improvements in neuropathology compared with SalUCO. At present, there are no studies of sexual dimorphisms in perinatal creatine levels nor treatment responses of creatine in the context of perinatal brain injury, which requires further investigation given the sex dependent vulnerability to HI injury at birth and the innate higher circulating creatine levels in female than in male subjects.^50^ Last, the optimal duration and timing of creatine preloading remains unknown. We demonstrated that in fetal sheep, 6 to 10 days of continuous intravenous creatine infusion at 6 mg/kg/h elicits an arterial plasma creatine concentration of around 400 to 600 μM and a ~22% increase in cerebral creatine levels, which provided prophylactic efficacy from asphyxic brain injury.^7^ At this stage, we cannot be certain about how the fetal i.v. creatine infusion dose in sheep translates to an effective enteral supplementation regimen provided to human pregnant mothers.

## Conclusions

Fetal creatine supplementation reduced seizure burden, improved EEG recovery, and reduced indices of cortical and subcortical histopathology following mild to moderate asphyxial encephalopathy, induced by UCO, in late gestation fetal sheep. These findings suggest creatine is an efficacious treatment for mild to moderate HIE and contribute to a growing body of evidence to support translational studies of maternal dietary creatine supplementation during human pregnancy to protect the newborn from adverse neurological outcomes.

## Author Contributions

N.T.T., S.J.E., R.J.S., D.W.W., and R.G. contributed to the conception and design of the study; N.T.T., S.J.E., S.B.K., J.S., H.L., G.R.P., R.J.S., S.J.E., D.W.W., and R.G. contributed to the acquisition and analysis of data; N.T.T., S.J.E., S.B.K., J.S., H.L., G.R.P., R.J.S., S.J.E., D.W.W., and R.G. contributed to drafting the text or preparing the figures.

## Potential Conflicts of Interest

The authors declare no commercial or financial relationships that could be construed as a potential conflict of interest. S.J.E. does serves as a member of the Scientific Advisory Broad on creatine in health and medicine (AlzChem LLC.). However, the company was not involved in the design or dissemination of this study.

## Supporting information


**Table S1.** Classification of EEG Background Activity Adapted from Murray et al. (2009).^1^
**Table S2.** Immunohistochemistry Protocol.
**Table S3.** Fetal Characteristics at Post‐Mortem.
**Table S4.** Baseline (−24 hours up to 0 hour) Data for Systemic Physiology and Neurophysiology Prior to UCO.
**Figure S1.** Example real‐time physiological recording.
**Figure S2.** Fetal systemic physiology and neurophysiology are unaffected with prolonged continuous creatine infusion.
**Figure S3.** Arterial blood gas and chemistry after creatine infusion.
**Figure S4.** Arterial blood gas and chemistry after UCO.
**Figure S5.** Prophylactic creatine supplementation alters the relative spectral power of frequency bands immediately upon UCO induction and after UCO.

## Data Availability

All data are available in the main text or the supplementary materials. The data from this study are available from the corresponding author upon reasonable request.
