# Supplementary material for: Prophylactic Fetal Creatine Supplementation Improves Post‐Asphyxial EEG Recovery and Reduces Seizures in Fetal Sheep: Implications for Hypoxic–Ischemic Encephalopathy
Source: Ann Neurol. 2024 Dec 7;97(4):673–87. doi: 10.1002/ana.27150 (PMC11889532; doi:10.1002/ana.27150)
Supplement: Supplementary file 1 — Table S1. Classification of EEG Background Activity Adapted from Murray et al. (2009). 1 Table S2. Immunohistochemistry Protocol. Table S3. Fetal Characteristics at Post‐Mortem. Table S4. Baseline (−24 hours up to 0 hour) Data for Systemic Physiology and Neurophysiology Prior to UCO. Figure S1. Example real‐time physiological recording. Figure S2. Fetal systemic physiology and neurophysiology are unaffected with prolonged continuous creatine infusion. Figure S3. Arterial blood gas and chemistry after creatine infusion. Figure S4. Arterial blood gas and chemistry after UCO. Figure S5. Prophylactic creatine supplementation alters the relative spectral power of frequency bands immediately upon UCO induction and after UCO. [file ANA-97-673-s001.docx]

**LIST OF SUPPLEMENTARY MATERIALS**

**Supplementary Materials and Methods**

**Table S1.** Classification of EEG Background Activity adapted from (Murray et al., 2009) (1)

**Table S2**. Immunohistochemistry protocol

**Table S3.** Fetal characteristics at post-mortem.

**Table S4.** Baseline (-24 h up to 0 h) data for systemic physiology and neurophysiology prior to UCO

**Figure S1**. Example real-time physiological recording

**Figure S2.** Fetal systemic physiology and neurophysiology are unaffected with prolonged continuous creatine infusion

**Figure S3.** Arterial blood gas and chemistry after creatine infusion

**Figure S4.** Arterial blood gas and chemistry after UCO

**Figure S5.** Prophylactic creatine supplementation alters the relative spectral power of frequency bands immediately upon UCO induction and after UCO

**Supplementary Materials and Methods**

*Animal welfare*

To align with the ethical use of laboratory animal experiments, historical physiological recordings and samples of vehicle sham fetuses of similar gestational age were used as the saline control group (SalCon; *n*=7) – these data were obtained under approved ethics (MMCA/2017/13). Pregnant ewes carrying singleton fetuses were transported to the Monash Medical Centre animal house 6-9 days prior to sterile surgery and allowed to habituate to the environment. Ewes were housed with 12-hour light/dark cycle (0800-2000), allowed access to food and water *ad lib*, were housed in the presence of other ewes and cage cleaned daily. Food and water intake, general wellbeing and urine and excrement were monitored daily.

*Sterile surgical procedure*

Surgery was performed at 118 dGA under strict aseptic conditions. The ewe’s right or left flank and underside neck region was shaved. After the ewe was initially anaesthetized with sodium thiopentone (20 mg/kg), the ewe was intubated with an endotracheal tube (size 8.0) for positive pressure ventilation (10-12 breaths/min; 5-8 L/min). The ewe was repositioned to the supine position and the ewe’s lower abdomen, neck and flank wool was shaved and thoroughly cleaned with 3 washes of aqueous chlorhexidine gluconate (0.5% w/v in 70% ethanol; Jurox, NSW, Australia), followed by 3 washes with betadine surgical scrub (10% w/v povidone-iodine; Perrigo, Australia). Prophylactic antibiotics (100 mg/ml ampicillin, Austrapen, Alphapharm Pty Ltd, Australia; and 100 mg/ml oxytetracycline hydrochloride, Engemycin-100, Coopers Animal Health, Australia) were administered by intravenous injection prior to the start of sterile surgery. Ewe was carefully transferred to the surgical table and limbs tied down. The physiological status of the ewe was monitored continuously and recorded via a three-way ECG, PQRS wave and heart rate; a capnograph, airway end tidal CO_2_ and a pulse oximeter, arterial oxygen saturation and pulse rate. A final flooding of betadine antiseptic solution (10% w/v povidone-iodine; Orion Laboratories Pty Ltd, Balcatta, WA) was sprayed over the ewe’s abdomen and the midline incision site was wiped by 10% aqueous hibitane solution prior to commencement of surgery.

At the end of surgery, an angiocathether was inserted into the maternal left jugular vein for administration of antibiotics. At the end of all surgical procedures, anesthesia gas was withdrawn. All incision sites were irrigated with Marcaine with adrenaline (0.5% Bupivacaine hydrochloride and adrenaline acid tartrate) for topical analgesia, and then sprayed with betadine antiseptic solution (10% w/v povidone-iodine). The maternal abdominal incision was covered with a sterile cotton pad which was removed 24 h post-surgery. The ewe was covered with netting (Surgifix, size 7, Australian Home Healthcare, Australia) from the neck to hind and all fetal and maternal catheters, probes, electrode leads were placed in plastic bags and attached to the netting. The ewe was then provided with a fentanyl patch for analgesia (75 µg/h, Durogesic, Sandoz, Canada) which was placed on the underside of the inner left thigh. The ewe was then disconnected from the ventilator and transferred to a mobile trolley in the upright position. Extubation only occurred once the ewe’s normal swallowing reflex returned.

*Post-operative care and physiological recordings set up*

Post-operative care for the ewe consisted of daily intravenous administration of oxytetracycline (500 mg in 5 mL; IV, Engemycin-100) for 3 days. Post-operative care for the fetus consisted of fetal (100 mg in 1 mL saline) and intra-amniotic (400 mg in 4 mL saline) ampicillin during the post-operative 3-day recovery. After ~ 4 h post-surgery, at which the ewe was mobile and freely eating and drinking, one side of the pen was moved in to restrict movement of the ewe to allow for set up of physiological recordings. Fetal arterial catheters were continuously perfused with heparinised saline at 0.2 mL/min to maintain patency.

*Experimental recordings*

Fetal arterial and intraamniotic pressure was measured using disposable pressure transducers (DTX Plus, BD Medical Systems, Australia) connected to a multi-channel bridge amplifiers and digital converter program (Powerlab, ADInstruments, Australia). EEG and EMG leads were connected to bio amplifiers and then Powerlab. Fetal MABP was corrected for maternal movement by subtraction of amniotic pressure.

*Fetal behavioural states threshold calculation*

For the EEG threshold, the average EEG voltage in 6-8 manually selected 10 min selections occurring during periods of high and low voltage EEG (HV, LV) activity was calculated; the voltage threshold was then calculated as the average voltage between the high and low voltage. For the EMG threshold, the average EMG voltage in 6-8 manually selected 10 min selections occurring during periods of high and low EMG activity was calculated; EMG activity was scored as high if the voltage amplitude was higher than 20% of the voltage range between low and high EMG activity.

*Immunohistochemistry protocol and analysis*

As previously described in (10), for each animal, an 8 µm thick rostral and caudal section was selected corresponding to section 720 and section 1120 according to the Michigan State University Sheep Atlas per immunohistochemical marker. Immunohistochemistry sections were first dewaxed, rehydrated and then washed in phosphate buffered saline (PBS; pH 7.4) and prepared for individual protocols for histochemical staining. After antigen retrieval was conducted, slides were rinsed with PBS and blocked for endogenous peroxidases and then blocked for non-specific binding. Slides were then incubated overnight at 4°C in the presence of primary antibodies according to **Table S2**. Slides were washed in PBS and incubated in secondary biotinylated IgG antibody raised in corresponding species according to **Table S2**. The slides were washed in PBS and incubated with avidin-biotin complex (ABC Elite kit; 1:200 in PBS; Vectastain^®^, Vector Laboratories, UK) for one hour and visualized with 3,3’-diamniobenzidine solution (DAB; 1 tablet in 10 mL dH_2_O; MP Biomedicals, Australia). All slides were dehydrated, then placed in xylene for cover-slipping using DPX (Merck, Germany). Negative control sections included in each staining protocol, where the primary antibody was omitted, did not have positive cellular staining.

For each immunopositive cell density quantification within the Cortex and IGWM, two fields of views (FOV; 410 µm x 320 µm) were analysed per gyri; as there were no regional differences between gyri, all four FOVs were averaged and expressed as cells per field. Immunopositive cell density quantification within the PVWM was conducted using three FOVs. Cortex, IGWM and PVWM were assessed in both rostral (frontal) and caudal (parietal) sections and all FOVs across sections were averaged. For the hippocampus, one FOV was placed in the CA1&2, CA3, CA4 and DG region; there were no regional differences in cell density within the hippocampal CA regions and thus all four FOVs were averaged and expressed as cells per field of the hippocampal CA region. For quantitative assessments of TUNEL-positive cell density specifically, regions of interest were manually outlined using Aperio Image Scope (Leica Biosystems, Germany) and the TUNEL-positive cells were manually counted within the regions and expressed as cells/mm^2^.

*Saline Control fetuses*

Sterile surgery for SalCon fetuses was undertaken at 122-124 dGA, 3-5 days later than the CrUCO and SalUCO groups. This allowed us to reduce the use of animals, in accordance with the Australian code for the care and use of animals for scientific purposes, by using this control group for multiple contemporaneous studies. SalCon fetuses underwent the same instrumentation, received the same analgesia and medications and showed no differences in fetal cardiovascular, neurophysiological or behavioral differences prior to UCO compared to the UCO groups (**Table. S4**). Moreover, fetal growth (body and brain weights, **Table. S3**) did not differ between the SalCon and UCO groups. Therefore, the slightly later surgery age is unlikely to compromise the use of the physiological recordings and samples of these fetuses as this study’s sham vehicle control group.

**Table S1. Classification of EEG Background Activity, adapted from (1)**

| **Grade** | **Findings** | **Description** |
| --- | --- | --- |
| 0 | Normal EEG findings | Continuous background pattern with normal physiologic features such as anterior slow waves, clearly defined sleep state cycling (SSC) |
| 1 | Normal/mild abnormalities | Continuous background pattern with slightly abnormal activity (e.g., mild asymmetry, mild voltage depression with poorly defined SSC) |
| 2 | Moderate abnormalities | Discontinuous activity with no clear SSC, or clear asymmetry or asynchrony, mild interburst intervals present |
| 3 | Major abnormalities | Discontinuous activity severe attenuation of background patterns, or no SSC with major interburst intervals |
| 4 | Inactive EEG findings | Isoelectric and background activity of <10 μV |

**Table S2. Immunohistochemistry protocol**

Summary of finalized protocols for immunohistochemistry stains conducted. **BSA:** bovine serum albumin; **GFAP:** Glial fibrillary acidic protein; **IBA-1:** Ionized calcium binding adaptor molecule-1; **PBS:** phosphate buffer.

| **Antibody** | **Detects** | **Antigen retrieval** | **Primary Antibody (1°Ab)** | **1°Ab diluent** | **[1°Ab]** | **Secondary Antibody (2°Ab)** | **2°Ab diluent** | **[2°Ab]** |
| --- | --- | --- | --- | --- | --- | --- | --- | --- |
| NeuN | Mature neurons | Citrate Buffer (10 mM Tri-sodium citrate in dH2O, pH 6.0; Sigma Aldrich) | Mouse anti-NeuN (Millipore, Germany; CAT#: MAB377) | DAKO diluent (Dako, USA) | 1:500 | Goat biotinylated anti-mouse IgG (Vector Labs, USA) | PBS | 1:200 |
| IBA-1 | Microglia | Citrate Buffer | Rabbit anti-IBA-1 (Wako Chemicals, USA; CAT#: 019-19741) | 1% BSA PBS | 1:1000 | Goat biotinylated anti-rabbit IgG (Vector Labs, USA) | 1% BSA in PBS | 1:200 |
| GFAP | Astrocytes | Proteinase K (Promega, USA) | Rabbit anti-GFAP (Dako, USA; CAT#: Z033401-2) | DAKO diluent | 1:500 | Goat biotinylated anti-rabbit IgG | PBS | 1:200 |

**Table S3. Fetal characteristics at post-mortem.**

Data expressed as mean±SD and analysed by One-Way ANOVA for parametric data or Kruskal-Wallis test for non-parametric data.

|  | **SalCon** | **SalUCO** | **CrUCO** |  |
| --- | --- | --- | --- | --- |
| Number (*n*) | 7 | 7 | 7 |  |
| Male:Female | 5:2 | 4:3 | 6:1 | ***P-*value** |
| Body weight (kg) | 4.73±0.61 | 4.45±0.98 | 4.41±0.64 | 0.696 |
| Brain weight (g) | 51.75±3.91 | 50.64±3.15 | 48.75±19 | 0.365 |
| Brain:Body ratio | 9.36±4.47 | 11.77±2.25 | 11.18±1.32 | 0.296 |
| Heart weight (g) | 33.33±2.23 | 35.36±2.58 | 34.90±1.23 | 0.781 |
| Heart:Body ratio | 7.05±0.36 | 8.02±0.31 | 7.80±0.31 | 0.113 |
| Liver weight (g) | 127.3±8.98 | 123.9±8.33 | 145.2±9.66 | 0.254 |
| Right Kidney (g) | 12.38±1.18 | 12.81±1.43 | 15.22±1.40 | 0.287 |
| Left Kidney (g) | 12.53±1.15 | 13.33±1.01 | 14.77±0.90 | 0.316 |

**Table S4. Baseline (-24 h up to 0 h) data for systemic physiology and neurophysiology prior to UCO**

Average baseline data calculated between -24 h up to 0 h relative to UCO induction for all systemic physiology and neurophysiology measurements. Data are mean±SD. SalCon, *n*=7; SalUCO, *n*=7; CrUCO, *n*=7. Statistical analysis is repeated measures two-way ANOVA, main effects *P* values are presented. * shows significant differences (*P*<0.05).

|  | ***Group*** | | | | | ***Two-way ANOVA*** | | |
| --- | --- | --- | --- | --- | --- | --- | --- | --- |
|  | | **SalCon** | **SalUCO** | **CrUCO** | ***P_TIME_*** | | ***P_Group_*** | ***P_Interaction_*** |
| MABP (mmHg) | | 44.07±0.64 | 42.32±0.54 | 41.42±1.11 | 0.607 | | 0.295 | 0.204 |
| FHR (bpm) | | 162.6±6.69 | 161.1±6.18 | 163.6±4.24 | **0.004*** | | 0.919 | 0.275 |
| EMG (µV) | | 2.60±0.42 | 2.04±0.22 | 2.38±0.32 | **0.034*** | | 0.651 | 0.288 |
| EEG Power (dB) | | 19.02±0.55 | 18.61±0.55 | 16.13±0.34 | 0.370 | | 0.115 | 0.992 |
| EEG SEF (Hz) | | 9.60±0.73 | 9.36±0.33 | 9.19±0.28 | 0.258 | | 0.890 | 0.834 |
| HVEEG (%) | | 43.19±8.42 | 44.84±7.04 | 44.08±6.35 | 0.333 | | 0.974 | 0.816 |
| LVEEG (%) | | 56.81±8.42 | 55.16±7.04 | 55.92±6.35 | 0.333 | | 0.974 | 0.816 |
| HVEEG+EMG (%) | | 32.84±7.23 | 30.91±7.19 | 32.57±7.41 | **0.039*** | | 0.916 | 0.300 |
| LVEEG+LEMG (%) | | 27.14±8.46 | 29.23±5.96 | 32.15±7.24 | 0.526 | | 0.454 | 0.398 |
| LVEEG+EMG (%) | | 29.63±4.67 | 33.62±7.37 | 23.77±8.50 | 0.470 | | 0.290 | 0.552 |
| Delta-band (%) | | 71.04±0.90 | 71.38±0.85 | 73.03±0.74 | 0.329 | | 0.820 | 0.966 |
| Theta-band (%) | | 13.26±0.54 | 13.20±0.39 | 12.14±0.39 | 0.122 | | 0.488 | 0.984 |
| Alpha-band (%) | | 7.98±0.53 | 8.00±0.38 | 8.37±0.43 | 0.245 | | 0.925 | 0.990 |
| Beta-band (%) | | 6.80±0.62 | 6.48±0.43 | 5.55±0.30 | 0.250 | | 0.653 | 0.809 |


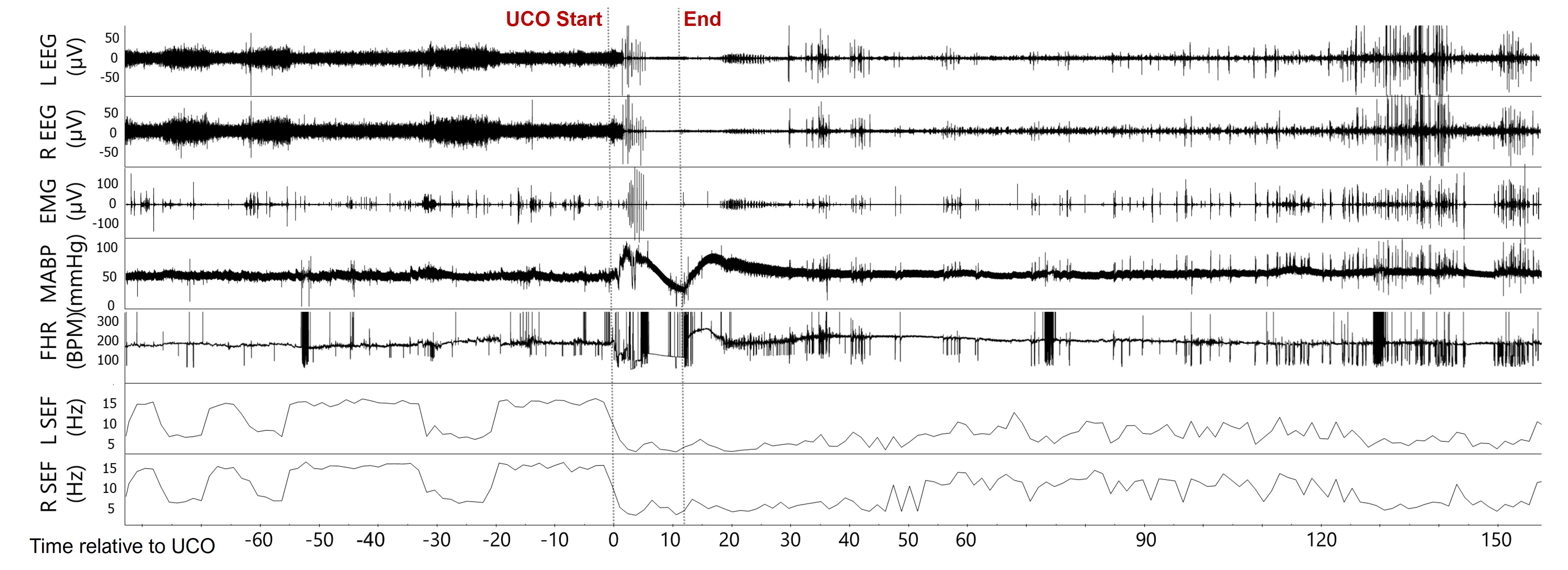


**Figure S1.** **Example real-time physiological recording**

Example real-time monitoring of electroencephalography (EEG) from the left (L) and right (R) cortical hemispheres, nuchal electromyography (EMG), mean arterial blood pressure (MABP), fetal heart rate (FHR), and EEG spectral edge frequency (SEF). Vertical lines indicate UCO onset and release (end); *x*-axis is time in minutes relative to onset of UCO.





**Figure S2. Fetal systemic physiology and neurophysiology are unaffected with prolonged continuous creatine infusion**

Systemic physiological recordings of **(A)** mean arterial blood pressure (MABP) and **(B)** fetal heart rate (FHR), and neurophysiological recordings of **(C)** nuchal electromyography (EMG) and cortical electroencephalography (EEG) **(D)** power and **(E)** spectral edge frequency (SEF) following continuous saline or creatine infusion. **(F)** EEG trace was divided into component frequency bands of slow **(G)** delta and **(H)** theta bands and fast **(I)** alpha and **(J)** beta bands following continuous saline or creatine infusion. Grey dotted line indicates start of allocated treatment infusion. All data (**A-E&G-J**) are one-hour epochs and presented as mean±SD. Two-way repeated measures ANOVA; *P*<0.05. SalUCO, red, *n*=7; CrUCO, blue, *n*=7

**Figure S3. Arterial blood gas and chemistry after creatine infusion**

Arterial blood gas measurements prior to umbilical cord occlusion (UCO); *x*-axis is time relative to start of saline/creatine infusion indicated by grey line. Measurements of pH, partial pressure of carbon dioxide (PaCO_2_), partial pressure of oxygen (PaO_2_), oxygen saturation (SaO_2_), glucose, lactate, base excess and bicarbonate (HCO_3_^-^). Data are mean±SD; SalUCO, *n*=7; CrUCO, *n*=7. Two-Way ANOVA repeated measures; *P*<0.05.





**Figure S4. Arterial blood gas and chemistry after UCO**

Arterial blood gas measurements after UCO and reperfusion (R); *x*-axis is time relative to UCO induction indicated by shaded grey area. Measurements of pH, partial pressure of carbon dioxide (PaCO_2_), partial pressure of oxygen (PaO_2_), oxygen saturation (SaO_2_), glucose, lactate, base excess and bicarbonate (HCO_3_^-^). Data are mean±SD; SalUCO, *n*=7; CrUCO, *n*=7. Two-Way ANOVA repeated measures; Bonferroni’s post-hoc test; and unpaired Student’s *t­*-test at timepoint +24 h. *P**<0.05.





**Figure S5. Prophylactic creatine supplementation alters the relative spectral power of frequency bands immediately upon UCO induction and after UCO**

Neurophysiological recordings of EEG frequency separation into component frequency bands from slow **(A)** delta and **(B)** theta bands and fast **(C)** alpha and **(D)** beta bands. **(i)** Dashed boxes are measurements during UCO; data are 20 s epochs and presented as delta (Δ) change from baseline. **(ii)** Data analysis during early (0-6 h), intermediate (6-24 h) and late (24-72 h) recovery periods are one-hour epochs. All data are presented as mean±SD. x-axis is time relative to UCO indicated by a grey bar. Two-way repeated measures ANOVA; Tukey’s post-hoc test. # denotes significant differences (P<0.05) between SalCon and SalUCO; $ denotes significant differences (P<0.05) between SalCon and CrUCO; * denotes significant differences (P<0.05) between SalUCO and CrUCO. SalCon, grey, n=7; SalUCO, red, n=7; CrUCO, blue, n=7.
